# Establishment of immortalized murine mesothelial cells and a novel mesothelioma cell line

**DOI:** 10.1007/s11626-015-9885-z

**Published:** 2015-04-15

**Authors:** Walter Blum, László Pecze, Emanuela Felley-Bosco, Janine Worthmüller-Rodriguez, Licun Wu, Bart Vrugt, Marc de Perrot, Beat Schwaller

**Affiliations:** 1 Department of Medicine, University of Fribourg, Route Albert-Gockel 1, 1700 Fribourg, Switzerland; 2 Laboratory of Molecular Oncology, Clinic of Oncology, University Hospital Zürich, Haeldeliweg 4, 8044 Zurich, Switzerland; 3 Thoracic Surgery Research Laboratories, Division of Thoracic Surgery, Toronto General Hospital, University Health Network, University of Toronto, Toronto, ON Canada; 4 Institute of Surgical Pathology, University Hospital Zürich, Zürich, Switzerland

**Keywords:** Mesothelioma, Asbestos, Mouse cell lines, Cell models, Immortalized, SV40

## Abstract

Mesothelial cells are susceptible to asbestos fiber-induced cytotoxicity and on longer time scales to transformation; the resulting mesothelioma is a highly aggressive neoplasm that is considered as incurable at the present time Zucali *et al.* (Cancer Treatment Reviews 37:543–558, 2011). Only few murine cell culture models of immortalized mesothelial cells and mesothelioma cell lines exist to date. We generated SV40-immortalized cell lines derived from wild-type (WT) and neurofibromatosis 2 (merlin) heterozygote (Nf2+/−) mice, both on a commonly used genetic background, C57Bl/6J. All immortalized mesothelial clones consistently grow in DMEM supplemented with fetal bovine serum. Cells can be passaged for more than 40 times without any signs of morphological changes or a decrease in proliferation rate. The tumor suppressor gene *NF2* is one of the most frequently mutated genes in human mesothelioma, but its detailed function is still unknown. Thus, these genotypically distinct cell lines likely relevant for malignant mesothelioma formation are expected to serve as useful in vitro models, in particular to compare with in vivo studies in mice of the same genotype. Furthermore, we generated a novel murine mesothelioma cell line RN5 originating from an Nf2+/− mouse subjected to repeated crocidolite exposure. RN5 cells are highly tumorigenic.

## Introduction

Malignant mesothelioma (MM) is an aggressive form of cancer with an average survival time of about 1 yr; up to date, conventional therapies have proven to be rather unsuccessful (Rascoe *et al.* 2012). Tumors are derived from transformed mesothelial cells, a cell type that is covering the internal cavities of the pericardium, peritoneum, and the pleura. The mesothelium is a cell monolayer consisting of flat cells, forming the barrier between the tunica mucosae parietalis and visceralis. Pleural MM is linked in up to 80% of the cases with asbestos exposure (Carbone *et al.* 2011). Besides exposure to asbestos fibers leading to MM formation, other etiologies including genetic disposition and, rarely, ionizing radiation were found associated to MM (Carbone *et al.* 2011; Chirieac *et al.* 2013). In addition, SV40 oncoproteins [large T and small t antigen (TAg and tag, respectively)] together with asbestos fibers were demonstrated to be co-carcinogenic (Bocchetta *et al.* 2000; Kroczynska *et al.* 2006). Animal models to study MM were long hampered due to the slow time-course between asbestos exposure and MM formation, a period that ranges from 20 to 40 yr in humans (Bianchi *et al.* 1997). Exposure of wild-type (WT) mice to asbestos fibers, even at high doses, results in the formation of MM only in a small fraction of animals (<33%) (Altomare 2005). Mutations in the *NF2* gene have been found in about 40% of human mesothelioma (Bianchi *et al.* 1995; Sekido *et al.* 1995; Deguen *et al.* 1998), and the influence of Nf2 heterozygosity has been investigated in mice; Nf2 heterozygosity was shown to result in MM at high frequency (85%) within 1 yr after repetitive asbestos exposure (Altomare 2005). In another transgenic mouse model, SV40 TAg expression driven by the mesothelin promoter leads to asbestos-induced MM in <1 yr (Robinson *et al.* 2006). However, for many aspects of MM research, in particular for the investigation of the early steps of mesotheliomagenesis, cell lines are of utmost importance, providing relatively inexpensive model systems with high throughput capacities and reducing the need for animal experiments. For the investigation of the early steps of MM formation, immortalized cell lines derived from primary mesothelial cells are the tools of choice. The most used immortalized human mesothelial cell lines are MeT-5A immortalized with SV40 (Ke *et al.* 1989) and LP9 cells immortalized with TERT1, the catalytic component of telomerase (Connell and Rheinwald 1983). Immortalized mesothelioma and mesothelial cells of mouse origin include cells isolated from the ascites of asbestos-exposed wild-type C57Bl/6J or BALB/c mice [40, 40L, AE17, AK7 (Cordier Kellerman *et al.* 2003), and AB12 (Davis *et al.* 1992)], SV40 TAg transgenic mice [MexTAg, line names TGM299h, TGM304i, TGM270i, and TGM266i (Robinson *et al.* 2006), conditional Nf2−/− mice (Jongsma *et al.* 2008)], and spontaneously immortalized cells obtained after prolonged culturing of primary mouse mesothelial cells (Sherwood *et al.* 2008). Here, we established SV40-immortalized mesothelial cells of WT mice and immortalized cells derived from (Nf2+/−) mice; all lines stem from mesothelial cells isolated from mice with C57Bl/6J background. We expect the latter cell lines to serve as valuable models to investigate in further detail the role of Nf2 (merlin) in vitro and to complement in vivo studies carried out in Nf2+/− mice. Immortalized mesothelial cell lines were not tumorigenic, but showed anchorage-independent growth in the spheroid formation assay, thus allowing to use them as 3D cell culture models (Smalley *et al.* 2006). Since the role of SV40 in MM is still controversially discussed in the mesothelioma field (Carbone *et al.* 2003), the different immortalized mesothelial cell lines also provide a model to investigate the effect of inactivating p53 in different genotypes (WT, Nf2+/−). Finally, a novel murine mouse mesothelioma cell line RN5 was generated from a tumor that had developed in a crocidolite-exposed Nf2+/− mouse; this line shows high tumorigenicity in vivo and persistent growth in vitro.

## Materials and Methods

### *Isolation of mouse primary mesothelial cells and mesothelioma cells from a NF2+/− mouse.*

Mesothelial cells were isolated from 4 to 6 months old WT and Nf2+/− mice, all on a C57Bl/6J background using a previously described protocol (Bot *et al.* 2003). Briefly, mice were killed and the peritoneal cavities were exposed by incision and removal of the fur. The peritoneal cavities were washed with injecting approximately 50 ml of phosphate-buffered saline (PBS) (Sigma, St. Louis) via a syringe equipped with a 25G × 5/8″ needle (BD microlance 3, Becton Dickinson AG, Allschwil, Switzerland) using a peristaltic pump and a second needle (23G, 0.6 × 25 mm) to allow exit of the PBS solution. Perfusion was maintained until the exiting PBS solution was clear, i.e., devoid of mobile and poorly attached cells. Residual PBS was aspired with a syringe, and the peritoneal cavity was filled with 5 ml of 0.25% Trypsin/EDTA solution (Gibco, Basel, Switzerland). The body temperature of mouse corpses was maintained at around 37°C for 5 min via an infrared heating lamp. The suspension containing the detached cells was collected with a syringe; cells were centrifuged for 10 min at 300×*g*. Cells mostly comprising primary mesothelial cells were grown in modified Connell’s medium composed of Dulbecco’s modified Eagle’s medium (DMEM)/F12 + GlutaMax (Gibco), 15% fetal calf serum (FCS), 0.4 μg/ml hydrocortisone, 10 ng/ml epidermal growth factor, 1% ITS (insulin, transferrin, selenium), 1 mM sodium pyruvate, 0.1 mM beta-mercaptoethanol, 1% non-essential amino acids, 1% penicillin–streptomycin, and 2% Mycokill (PAA) (Connell and Rheinwald 1983). The *Nf2* alleles (WT or mutated) were genotyped using the common forward primer (NF2_FW 5′-GGGGCTTCGGGAAACCTG G-3′), and either NF2_RV WT (5′-GTCTGGGAAGTCTGTGGAGG-3) or NF2_RV mutant (5′-CTATCAGGACATAGCGTTGG-3′) primers. The cell line RN5 was isolated from an Nf2+/− mouse that was repeatedly injected with crocidolite starting at 8 wk of age (7 × 400 μg). Briefly, a clearly discernible tumor localized on the liver was dissected from the mouse 21 wk after the first injection. The tissue was incubated in a 0.25% Trypsin/EDTA solution for 10 min; tumor cells were dissociated by mild trituration and cultured in DMEM, 10% fetal bovine serum (FBS, Gibco, Basel, Switzerland), and 1% PS (100 U/mL penicillin and 100 μg/mL streptomycin).

### *Cell culture and growth curves.*

Primary mesothelial cells from the two genotypes (WT and Nf2+/−) were immortalized by transduction with SV40 small and large T antigen. Transduction was performed using lentivirus that were produced as previously described (Blum and Schwaller 2013). The transfer plasmid pCMV/TO SV40 was obtained from Addgene (# 22298). Primary cells were seeded at a density of 10,000 cells/cm^2^; 24 h post-seeding cells were transfected with lentivirus at an MOI of 5. Proliferation was monitored using the Incucyte Live Cell Imaging System (Essen Bioscience, Michigan). After passaging, cells were seeded at a very low density (1–2 cells/cm^2^) in 10-cm Petri dishes. Individual and well-delimited clones were picked and transferred into 96-well plates and frozen at passages 7–10 following standard protocols. 3-(4,5-Dimethylthiazol-2yl)-2,5-diphenyltetrazolium bromide (MTT) assay was performed as previously described (Blum and Schwaller 2013).

### *Western blot assay for mesothelin, SV40 large T-antigen, and β-actin.*

Cells were grown in 75-cm^2^ cell culture flasks (TPP, Trasadingen, Switzerland) until they reached nearly 100% confluence. Subsequently, cells were trypsinized and pelleted (500×*g*, 3 min), then washed three times with CMF-PBS. Proteins were extracted with RIPA buffer [50 mM Tris, 150 mM NaCl, 0.1% sodium dodecyl sulfate (SDS), 0.5% sodium deoxycholate, 1% Triton X-100, pH 7.4], incubated for 30 min on ice, and centrifuged 30 min at 17,000×*g* at 4°C, and the supernatant was collected. The DC assay (BioRad) was performed to quantify the proteins following the manufacturer’s protocol. Protein samples were separated on a 10% polyacrylamide SDS gel and transferred onto nitrocellulose membranes. Membranes were checked with Ponceau S staining for equal loading. Membranes were blocked with 5% milk PBS for 1 h at room temperature and incubated overnight at 4°C with antibodies recognizing mesothelin (dilution 1:2,000; Santa Cruz Biotechnology, Santa Cruz, CA, clone B-3), SV40 large T-antigen (1:2000; Santa Cruz Biotechnology, Pab101), and β-actin (1:20,000; Sigma-Aldrich, clone ac-74). Secondary biotinylated antibodies were used at a dilution of 1:20,000, and the ABC system (Vectastain, Vector Laboratories, Burlingame, CA) was applied. The HRP substrate (Millipore, Luminata Forte) was incubated for 3 min on the membrane and analyzed on a Western blot reader (FluorChem E System, Bucher Biotec, Basel, Switzerland).

### *Soft agar assay.*

The procedure of the soft agar assay was adapted from a previously described protocol (Provost *et al.* 2012). Shortly, 0.5% of base agar was prepared by melting 1% of agar (LuBioScience, Lucerne, Switzerland) in a microwave oven and adding of prewarmed (40°C) 2× RPMI completed with 20% FCS and antibiotics (penicillin-streptomycin) and allowed to equilibrate to 37°C. Equal volumes of the solutions were mixed to give final concentrations of 0.5% agar, 1× RPMI, and 10% FCS. One milliliter was added per well in six-well plates and set aside to solidify. Top agar was prepared by melting 0.7% agarose (PeqLab, Erlangen, Germany) in a microwave oven and cooled down to 40°C and then supplemented with an equal volume of 2× RPMI with 20% FCS also at approximately 40°C. Cells (5000) in a volume of 1 ml of 0.35% agarose in 1× RPMI + 10% FCS was added to the solidified agar. Plates were incubated for 17 d in a humidified incubator at 37°C with 5% CO_2_. Twice per week, the plates were supplied with 0.5 ml of cell culture medium.

### *Subcutaneous xenograft model.*

WT C57Bl/6J mice (6–8 wk old; *n* = 3–5 animals depending on the group) were inoculated s.c. with 4 × 10^5^ cells by injection of the cell suspension (50 μl in PBS containing 20% matrigel) into the right dorsal flank. Cells included immortalized mesothelial cells (iMeso-WT1 and iMeso-NF3), the MM cell line RN5, and the previously described murine MM cell line AK7 (Cordier Kellerman *et al.* 2003), the latter also on a C57Bl/6J background. Tumors were measured with a caliper weekly as soon as palpable; the volume was calculated using the formula *a* × *b* ^2^ × 0.5, where *a* is the large and *b* the small diameter of an ellipse. For the immunohistochemistry, deparaffinized sections were subjected to antigen retrieval using sodium citrate, pH 6, then were processed as previously described (Frei *et al.* 2011). Primary antibodies used were anti-wide spectrum cytokeratin and anti-vimentin (Abcam Ab9377, 1:100 and Ab92547, 1:500, respectively).

### *Immunofluorescence experiments.*

Cells were seeded on glass coverslips and incubated with the appropriate cell culture medium until a confluence of 70–90% was reached. Then, the cells were fixed with 4% PFA, permeabilized with 1% Triton X-100, and blocked with TBS solution containing donkey serum (10%). Cells were incubated overnight at 4°C with the primary antibodies at the indicated dilutions: mouse monoclonal anti-pan-cytokeratin, clone Lu-5 (BMA Biomedicals, Augst, Switzerland, T-1302; 1:500), or rabbit polyclonal anti-desmin (Sigma D8281; 1:500). After washing, the cell-containing coverslips were incubated with secondary antibodies for 1 h with either DyLight488-conjugated donkey anti-mouse IgG (Jackson Immunoresearch Laboratories, West Grove, PA; 1:1,000) or Cy3-conjugated donkey anti-rabbit IgG (Jackson Immunoresearch Laboratories; 1:1,000). The cells were counterstained with DAPI (Molecular Probes, Eugene, OR; 5 μg/ml) and mounted with Hydromount solution (National Diagnostics, Atlanta, GA). Images were acquired with a LEICA fluorescent microscope DM6000B (Wetzlar, Germany) equipped with a Hamamatsu camera C4742-95 (Bridgewater, NJ).

## Results

### *Immortalized primary mouse mesothelial persistently grow in vitro.*

**Figure 1.**
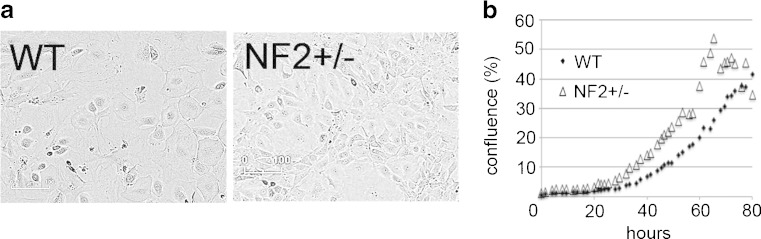
Morphology (*a*) and growth characteristics (*b*) of primary mesothelial cells isolated from WT and Nf2+/− mice, both grown in Connell’s medium. (*a*) Mesothelial cells from both genotypes show the typical “cobblestone-like” morphology. (*b*) Nf2+/− cells show slightly increased proliferation compared to WT cells; representative growth curves for *n* > 3 experiments are shown.

Primary mesothelial cells isolated from WT and Nf2+/− mice were initially grown in Connell’s medium, a medium previously shown to be optimal for mesothelial cell growth (Connell and Rheinwald 1983). Growth of cells from both genotypes was faster then in the medium used for the further experiments, i.e., in DMEM supplemented with 10% FBS (data not shown). Primary mesothelial cells from WT and Nf2+/− mice grown in DMEM showed the typical cobblestone-like morphology similar as, e.g., the human mesothelioma cell line ZL55 consisting of cells with an epithelioid morphology used in previous studies (Blum and Schwaller 2013) (Fig. 1a). As shown before for WT primary mouse mesothelial cells (Robinson *et al.* 2006), mesothelial cells from the two genotypes grew in vitro for approximately 8–10 passages, followed by a prolonged period of quiescence lasting for up to several weeks before finally dying.

**Figure 2.**
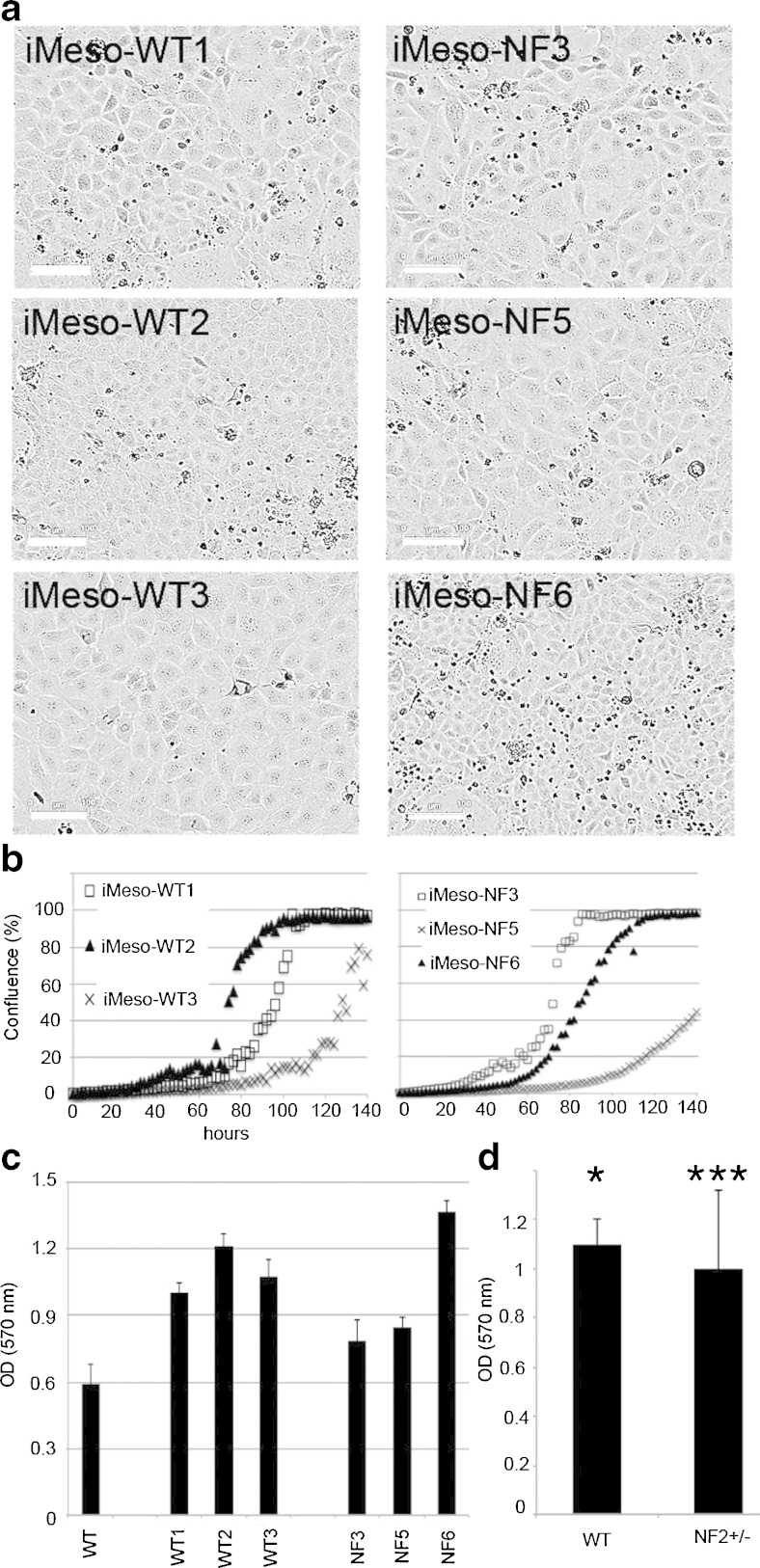
Immortalized mesothelial cell morphology and cell growth in DMEM + 10% FBS. (*a*) Brightfield images were taken with the Incucyte Live Cell Imaging system (FLR 10×) and show immortalized mesothelial cell clones derived from WT and Nf2+/− mice. The typical “cobblestone-like” morphology is conserved; all clones show a higher proliferation rate than their nonimmortalized primary counterparts (scale bar: 100 μm). (*b*) Representative Incucyte growth curves of WT and Nf2+/− clones. (*c*) MTT signals obtained at 144 h postseeding using all cells and cell lines as in Fig. 1 and 2*a*. (*d*) MTT data grouped for three clones each of the genotypes WT and Nf2+/− in comparison to the primary mesothelial cells (*p < 0.05; ***p < 0.0005).

Of note, primary mesothelial cells (initial density, 1500 cells/cm^2^) from Nf2+/− mice showed slightly enhanced proliferation compared to the cell lines generated from WT mice (Fig. 1b). From the initial batch of immortalized cells, by limited dilution, clones derived from three colonies for each genotype, likely originating from a single cell, thus representing single clones, were isolated, expanded, and stored for further usage. Unlike the primary mesothelial cells, SV40-immortalized mesothelial cell clones (iMeso-WT, iMeso-Nf2) grew rapidly in DMEM supplemented with 10% FBS (Fig. 2). Cells from both genotypes showed persistent cell proliferation even under low serum conditions (2%) and all survived for prolonged periods of time (>120 h) under very low serum conditions (0.5%), however without signs of proliferation evidenced by lack of mitotic cells (data not shown). All cultured mesothelial cells showed the typical cobblestone-like morphology like primary mesothelial cells from WT and Nf2+/− (Fig. 2a). All clones irrespective of genotype maintained their typical pavement-like morphology, and moreover, immortalized cell lines of both genotypes showed an increased cell proliferation rate when compared to the initial primary mesothelial cells (Fig. 2b) evidenced from real-time growth curves, as well as from the results of an independent proliferation assay, the MTT assay, carried out 144 h post-plating (Fig. 2c). Of note within one genotype (WT and Nf2+/−), growth rates of the various clones were considerably different, e.g., WT clones 1, 2, and 3 reached the plateau (close to 100% confluence) within 100, 80, and ≈140 h, respectively (Fig. 2b); similar proliferation rates were also observed for Nf2+/− clones. This was also confirmed using the MTT assay (Fig. 2c), when clones were grouped according to their genotype. No significant differences in growth rates were evident between the group of WT and NF2+/− clones (Fig. 2d). All SV40-immortalized mesothelial cell lines (clones) were cultured for more than 40 passages without showing any signs of a decreased growth and/or changes in cell morphology. A stock was produced at passage number 7–10 and frozen for long-term storage.

### *Characterization of primary and immortalized mesothelial cells.*

**Figure 3.**
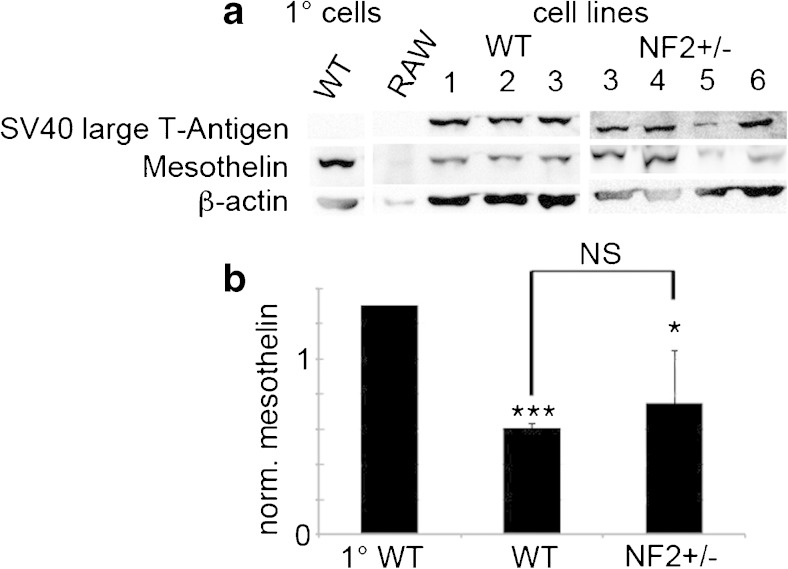
Western blot for SV40 large T antigen and mesothelin in WT and Nf2+/− cells in comparison of the primary immortalized clones from WT and Nf2+/− mesothelial cells. (*a*) Western blot signals from a representative experiment. The Western blot signals for β-actin were used for the normalization of the mesothelin expression levels. (*b*) Quantitative analysis of a representative Western blot (*n* = 3 samples per genotype, *p* < 0.05 grouped primary cells vs. grouped immortalized cell lines).

As expected, primary mesothelial cells of either genotype (WT, Nf2+/−), as well as the murine macrophage cell line RAW264.7 (Raschke *et al.* 1978), did not express TAg evidenced by Western blotting (Fig. 3a). Strong TAg expression was observed in all primary mesothelial cell clones transfected with the lentivirus coding for TAg/tag (Fig. 3). All cells (primary, immortalized) of mesothelial origin were clearly positive for mesothelin, a mesothelial cell-specific marker. The single positive band on the Western blots had a relative molecular mass (*M*_r_) of 69 kDa corresponding to the mesothelin precursor protein. Western blot quantification revealed decreased mesothelin levels in immortalized cell clones from both genotypes compared to primary cells WT mice (Fig. 3b). Immunohistochemical analysis showed homogeneous positive staining for desmin and mosaic-like expression for Pan-cytokeratin in all examined cell lines including the primary mesothelial cultures, the immortalized cells, and the tumor-derived RN5 cells. A typical example for RN5 cells is shown in Fig. 5c.

### *Primary mesothelial cells and SV40-immortalized mesothelial cells form colonies in the soft agar assay.*

**Figure 4.**
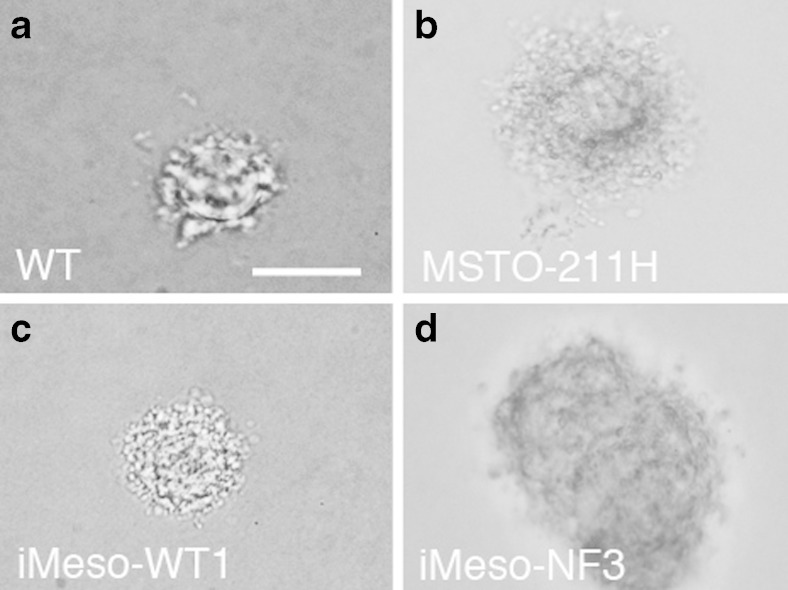
Colony formation assay. (*a*) Primary WT mesothelial cells were able to form colonies in plates with soft agar, as well as the human MM-derived cell line MSTO-211H (*b*) used as a positive control (derived from a biphasic mesothelioma). Selected colonies typically representing the morphological appearance of immortalized cells iMeso-WT1 (*c*), and iMeso-NF3 (*d*) are shown. *Scale bar*: 250 μm.

Since immortalized mesothelial cell clones of both genotypes (WT, Nf2+/−) also proliferated in low-serum (2%) conditions, a feature often observed in transformed cells, we investigated another aspect related to cell transformation, i.e., their ability to form anchorage-independent colonies in a semisolid medium. Cells from the various clones grew in soft agar, and images were taken after 17 d (Fig. 4). We then also tested primary mesothelial cells from WT mice; they were also able to form colonies of ≥250 μm diameter after 17 d of growth in anchorage-independent conditions (Fig. 4). These findings are in line with results on human primary mesothelial cells, where colony formation in semisolid medium has been reported before (La Rocca and Rheinwald 1985). Thus, anchorage-independent growth of primary mesothelial cells appears not to be restricted to cells of human origin; moreover, the soft agar assay does not allow to distinguish primary from immortalized or even transformed (RN5) cells. As a positive control, the human MM cell line MSTO-211H was used. The sole purpose of these experiments was to test whether or not murine mesothelial cells (primary, immortalized) have the capability of growing in soft agar in an anchorage-independent way. No attempt was made to correlate the mesothelial cell’s nature (primary vs. immortalized vs. transformed, from WT vs. transgenic mice) with the size of the colonies.

### *Morphological and growth characteristics of the novel RN5 cell line compared to AK7 and AB12 mouse mesothelioma cell lines.*

**Figure 5.**
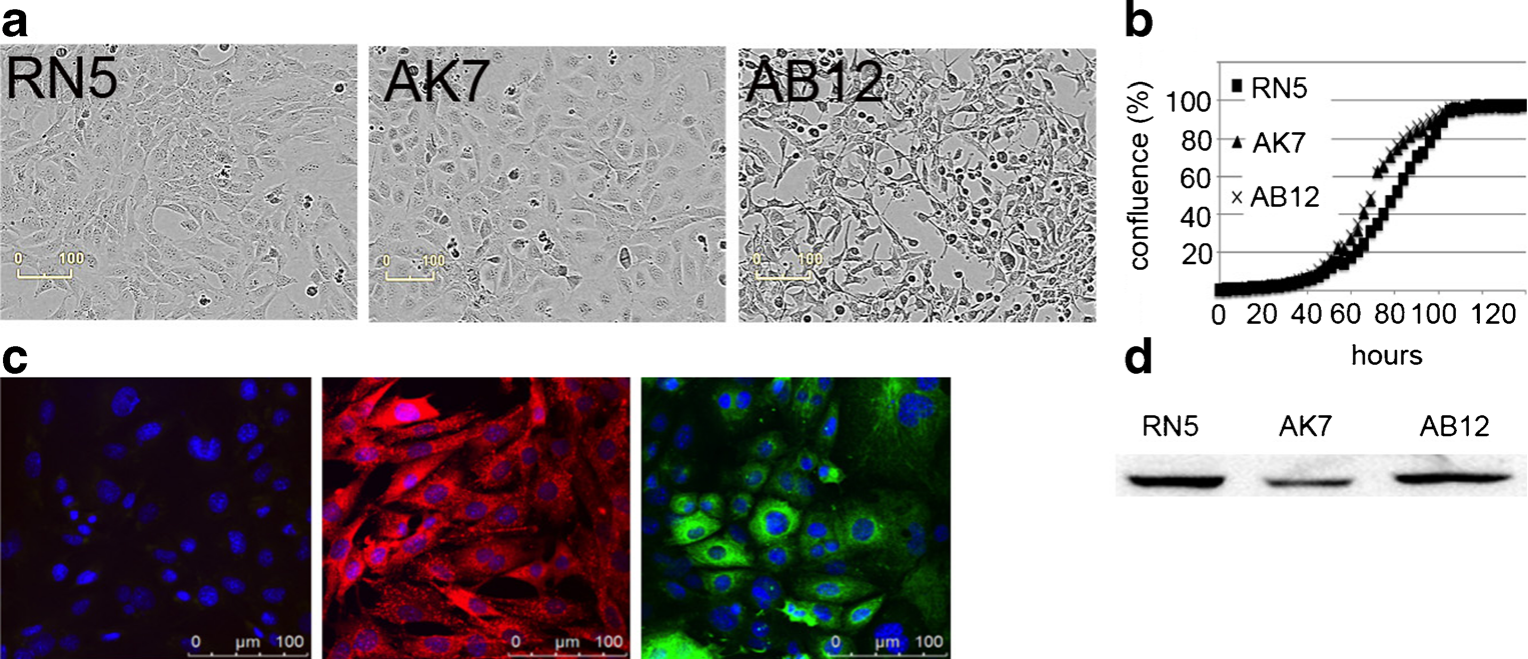
Morphology and growth of the murine mesothelioma cell lines RN5, AK7, and AB12 in vitro. (*a*) Epithelioid morphology of RN5 and AK7 compared to biphasic or mixed type morphology of AB12 cells. Cell size, morphology, and growth characteristics (*b*) are strikingly similar for RN5 and AK7 cells. (*c*) Immunofluorescene images of RN5 cells show a rather homogeneous staining for desmin (*red*). Pan-cytokeratin staining (*green*) shows a typical mosaic pattern; cell nuclei were labeled with DAPI (*blue*). (*d*) Western blot analysis (30 μg total protein/lane) showing the expression of mesothelin in the three murine mesothelioma cell lines RN5, AK7, and AB12.

We isolated a primary tumor obtained after repeated injection of asbestos fibers in the peritoneum of an Nf2+/− mouse. At the age of 29 wk, many tumors covered the spleen and the liver of this mouse. From a well-delimited piece of tumor tissue, the tumor cells were isolated by trypsinization of the tumor tissue followed by plating in DMEM. These cells, named RN5, have a similar morphology like AK7 cells in vitro, i.e., both cell lines show an epithelioid morphology, nearly as cobblestone-like as primary mesothelial cells (compare Fig. 5a and Fig. 1a). AB12 cells show sarcomatoid morphology in most cells and some epithelioid cells hinting towards a biphasic type. All three cell lines show fast proliferation and similar growth characteristics as evidenced by real-time imaging experiments (Fig. 5b).

### *RN5 cells form tumors in the subcutaneous xenograft model.*

**Figure 6.**
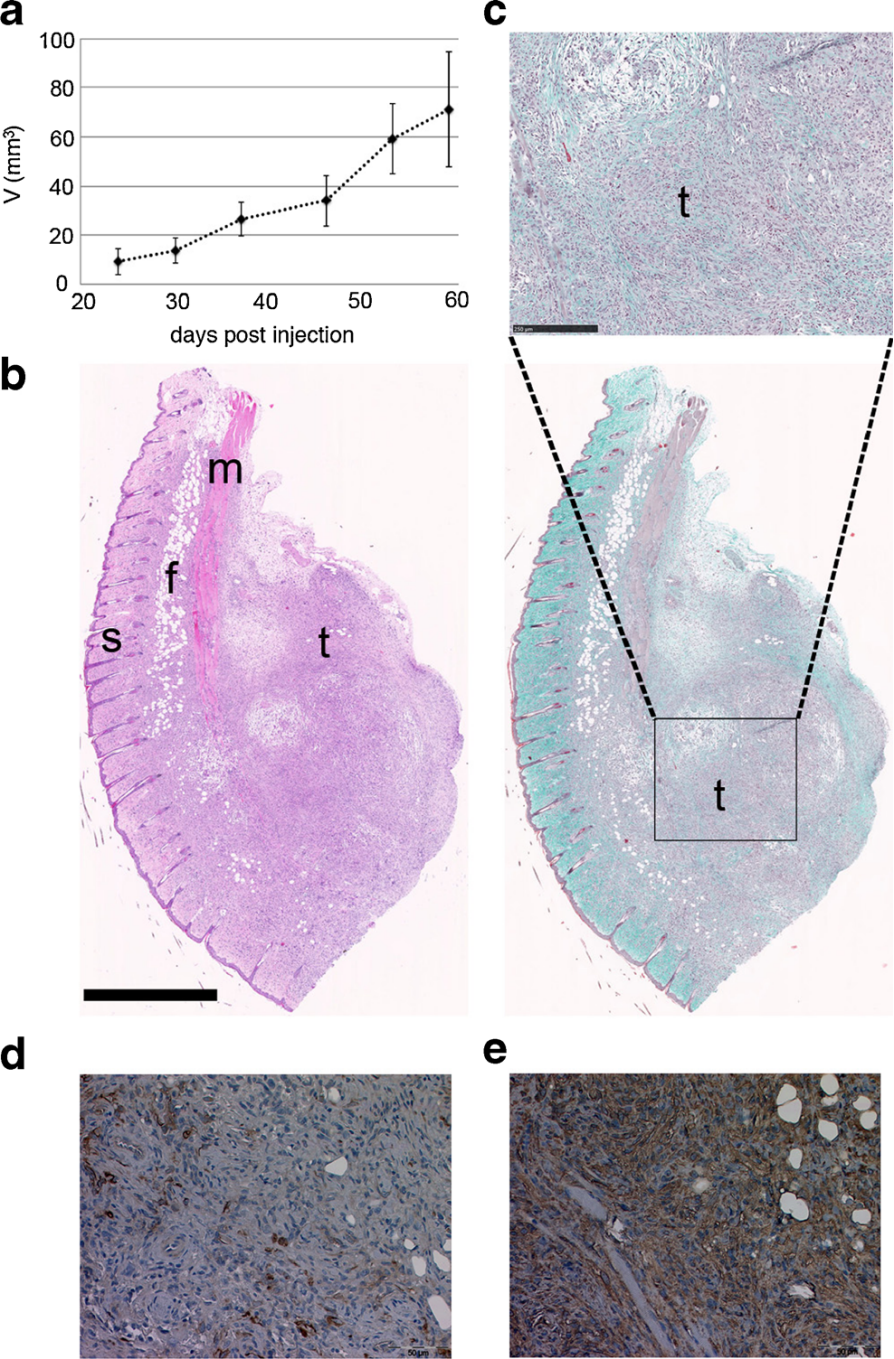
RN5 subcutaneous xenograft. (*a*) Xenograft growth monitored weekly 24 d after inoculation (*n* = 4 mice). (*b*) Histological sections of the xenograft stained by hematoxylin and eosin (*left*) and Goldner (*right*) showing the skin (*s*), the subcutaneous fat tissue (*f*), and muscle (*m*). *Scale bar*: 1 mm. (*c*) Magnification emphasizing tumor tissue (*t*) from the Goldner colored section. *Scale bar*: 250 μm. Immunohistochemical analysis showed expression of vimentin (*d*) with strong coexpression of pan-cytokeratin (*e*) in part of the tumor cell population.

Primary mesothelial cells from WT mice, immortalized cells iMeso-WT1, iMeso-NF3, and mesothelioma cells from the lines AK7 and RN5, all derived from a C57Bl/6J background, were injected (4 × 10^5^ cells) subcutaneously into the right flank of C57Bl/6J mice. Tumor formation was observed in all four mice injected with RN5 cells (Fig. 6a). No macroscopically evident (palpable) tumors were observed in mice injected with primary mesothelial cells of WT origin or with immortalized cells (iMeso). No tumors developed after injection of the selected number of AK7 cells; a significantly higher number (>2 × 10^6^ cells) has been reported to be required in order to induce macroscopic tumors in this experimental model (Cordier Kellerman *et al.* 2003). The tumor tissue originating from RN5 cells isolated 59 d after injection was white in color and rather solid at palpation. On histological sections subjected to a Goldner staining, a fibrous stroma was evident (Fig. 6c). Histological examination of tumors (Fig. 6b) derived from RN5 cells isolated 59 d after injection revealed a proliferation of atypical cells with a sarcomatoid morphology. The tumor was mainly confined to the subcutaneous tissue with infiltration of small clusters as well as individual cells into the dermis. Immunohistochemically, the tumor cells were positive for vimentin with strong coexpression of pancytokeratin in part of the cell population (Fig. 6d, e). No expression was seen for WT1 and calretinin (data not shown). The morphology combined with staining for vimentin and pancytokeratin is consistent with a sarcomatoid mesothelioma.

## Discussion

Cell culture systems in vitro represent helpful models to investigate tumor formation and, together with murine cancer models in vivo, have become a fundamental tool in Life Sciences (Balmain 2002). We generated simultaneously and on the same C57Bl/6J genetic background SV40-immortalized murine peritoneal mesothelial cell clones from two different genotypes: WT and Nf2+/−. This strategy offers several advantages in studies aimed to characterize, e.g., nanomaterial toxicity (asbestos, novel nanomaterials). The most obvious is that it allows characterizing the influence of Nf2 (merlin) without concerns about the influence of a genetic drift observed in inbred strains with mixed background or in C57BL/6 substrains (Zurita *et al.* 2011). Our murine cell lines derived from *Nf2* heterozygous mice provide a model system to investigate Nf2 (merlin) function and to possibly investigate the mechanisms leading to the inactivation of the nonmutated allele. Indeed, although Nf2-deficient murine cell lines are available (Jongsma *et al.* 2008), they are, in addition, also deficient for cyclin-dependent kinase inhibitor 2A (*Cdkn2a*) and, moreover, are on a mixed genetic background.

Mesothelial lines immortalized with SV40 T antigens have allowed highlighting the importance of p53 in maintaining genomic stability (Levresse *et al.* 2000; Pietruska and Kane 2007). We confirmed that SV40 T antigen expression, although accelerating the rate of the cell cycle, consistent with previous data (reviewed in An *et al.* 2012), is not sufficient to transform mesothelial cells (Cleaver *et al.* 2014). Therefore, they may constitute a suitable model to investigate early steps of mesothelial transformation, however also taking into consideration the limitations of such a model.

The establishment of the novel mouse mesothelioma cell line RN5 originated from a heterozygote Nf2+/− mouse on a C57Bl/6J background is expected to be useful also for in vivo investigations on (1) the modulation of tumor growth by decreased merlin levels (possibly linked to loss of heterozygosity), (2) the role of the immune system in asbestos-mediated mesothelioma development, and (3) the role of other stromal components in tumorigenesis. Tumorigenicity could be investigated in WT vs. the large variety of C57Bl/6J derived-mice deficient in stromal components. Moreover, RN5 is the first cell line from C57Bl/6J mice that is uniquely heterozygous for Nf2.

In conclusion, we have established new immortalized mouse mesothelial cell lines that provide model systems to study, e.g., mechanisms implicated in mesothelial transformation or to test for nanomaterial toxicity. We expect that these in vitro models will also help to reduce animal experimentation. The cell line RN5 was demonstrated to be fast and persistently growing in vitro and to be highly tumorigenic in syngeneic C57Bl/6J mice. These tumor cell-exposed mice are expected to retain a functional immune response. We foresee that this in vivo model will allow for testing putative therapeutic options against malignant mesothelioma.
